# Editorial: Pelvic floor disorders: a multidisciplinary issue

**DOI:** 10.3389/fsurg.2024.1400636

**Published:** 2024-03-22

**Authors:** Roberta Tutino, Francesco Pata

**Affiliations:** ^1^ Department of General and Emergency Surgery, AOU Città Della Salute e Della Scienza di Torino, Turin, Italy; ^2^ Department of Pharmacy, Health and Nutritional Sciences, University of Calabria, Rende, Italy

**Keywords:** pelvic floor disorders, rectal prolapse, prolapse, incontinence, pelvic floor rehabilitation, urinary incontinence, obstructed defecation, anismus

**Editorial on the Research Topic** Pelvic floor disorders: a multidisciplinary issue

Pelvic floor disorders encompass a wide range of pathologies and syndromes that significantly affect patients’ quality of life. When evaluating these patients, the assessment of the anterior, middle, and posterior compartments is necessary, as their involvement can be isolated or combined, leading to a mixed pattern of symptoms (1). Multidisciplinarity is essential to provide a comprehensive assessment. Moreover, functional disorders may not always correlate with anatomical abnormalities. The etiology is often multifactorial, involving anatomical defects in muscles and ligaments, or physiological factors such as denervation (2). Common medications and comorbidities affecting bowel function can confound the diagnostic process, along with gastrointestinal, neurological, psychiatric, endocrine, and metabolic disorders (3). A thorough investigation of all these aspects is necessary.

Pescatori et al. (4) underscored that conditions like rectocele and rectal internal mucosal prolapse, common indications for surgery, are just the “tip of the iceberg”. Anxiety, depression, anismus, neuropathy, middle-anterior prolapses, cystocele, prostatism, rectal hyposensation, irritable bowel disease, slow transit constipation, elithrocele, and solitary rectal ulcers can coexist in up to 66% of patients (4).

Several diagnostic approaches and treatment options are available, ranging from simple lifestyle modifications to advanced pharmacological and surgical interventions (5). Urinary incontinence and defecation disorders can coexist in prolapse, which can be classified as mono- bi-, or multicompartmental. While conservative treatments may suffice for smaller prolapses, surgery is typically indicated for complete prolapses.

In functional disorders, symptoms may not only improve but also worsen following anatomical correction, emphasizing the importance of offering a complete explanation to patients before any treatment and conducting a thorough evaluation to select the most appropriate therapy (6–8). Patients must informed about the possibility of recurrences and persisting pelvic floor symptoms (9, 10) to avoid unrealistic expectations. Rehabilitation programs must evaluate patient motivation, cooperation, and readiness to undertake intensive, prolonged therapy (11). Patients should understand their key role in reaching the target and the need for regular daily exercise. Close cooperation between referring physicians, therapists, and patients is crucial.

Factors such as female gender, potential obstetric trauma, wider pelvis, and age-related weakening of the pelvic floor may contribute to a poor urinary or defecatory function. Hence, a multidisciplinary evaluation involving proctologists, urologists, gynecologists, and pelvic floor rehabilitation specialists is imperative. Surgery and rehabilitation must act together to care for these patients.

Patients should undergo a holistic preoperative assessment, including a record of fecal incontinence, constipation, dysuria, urinary incontinence, pelvic organ prolapse and sexual troubles (12). A complete patient history (physiological, pathological, and pharmacological) is mandatory to identify limiting/confusing factors that can invalidate results and make treatments difficult to carry out correctly.

Advanced functional tests are recommended by gastroenterological guidelines (5), but they may be unnecessary with a thorough history and clinical examination. Some tests are often not available in all settings.

The British National Health System (NHS) mandates a rehabilitation treatment before any surgical intervention for pelvic floor functional disorders, a practice not universally adopted, with significant variability observed across different countries.

This editorial underscores the necessity of standardizing treatment protocols to facilitate actions by international scientific societies. It aims to foster dialogue and collaboration among specialists from diverse fields, thereby enhancing patient care in the field of pelvic floor disorders.

Accordingly, Molina et al. performed an observational study to investigate how the different pelvic disorders are associated with changes in quality of life (QoL). They administered a self-developed questionnaire on a cohort of 1,446 Spanish women investigating sociodemographic data, employment history and health status, lifestyle and habits, obstetric history, and health problems. They showed that pelvic organ prolapses, colorectal-anal symptoms, and urinary symptoms affect quality of life (QoL) globally and specifically. All categories of SF-12 questionnaire exhibit deterioration in patients with pelvic floor dysfunction, with vitality and emotional role being the most affected dimensions. The authors suggest that physical activity seems to be a “protecting factor” since active women show better results in terms of QoL.

The necessity to standardize the evaluation of pelvic floor muscle is emphasized by Huang et al., who conducted a systematic review to ascertain whether measurements of pelvic floor muscle alter across different body positions. The objective is to provide therapists with an accurate method for the evaluation and re-evaluation of functions, thereby mitigating biases associated with the patient's position.

Following these articles focusing on general scenarios, the work by O’Connor et al. offers a reflection on the management of fecal incontinence. The authors tried to verify if a positive response to PTNS (percutaneous tibial nerve stimulation) was followed by a positive response to SNS (sacral nerve stimulation). In their retrospective study, they confirmed the benefit of both treatments in fecal incontinence, but without a relation between responses. Some patients with a negative PTNS had a positive response to SNS.

Lastly, the benefit of a modified Altemeier procedure in the treatment of complete rectal prolapse was investigated by Wang et al. Since recurrences in perineal techniques are bothersome, introducing a muscular-serosal anastomosis has resulted in a recurrence rate of 1.54% vs. 26.47% with the traditional Altemeier. However, multicentric and randomized studies will be necessary to confirm these promising preliminary results.

Pelvic floor disorders pose numerous unanswered questions, and the efficacy of various treatments requires stronger evidence. This issue aims to provide insights into addressing some questions.
